# Inhibition of Proinflammatory Enzymes and Attenuation of IL-6 in LPS-Challenged RAW 264.7 Macrophages Substantiates the Ethnomedicinal Use of the Herbal Drug *Homalium bhamoense* Cubitt & W.W.Sm

**DOI:** 10.3390/ijms21072421

**Published:** 2020-03-31

**Authors:** Rungcharn Suksungworn, Paula B. Andrade, Andreia P. Oliveira, Patrícia Valentão, Sutsawat Duangsrisai, Nelson G. M. Gomes

**Affiliations:** 1 Department of Botany, Faculty of Science, Kasetsart University, Ngam Wong Wang Road, Chatuchak, Bangkok 10900, Thailand; b5310402969@gmail.com; 2 REQUIMTE/LAQV, Laboratório de Farmacognosia, Departamento de Química, Faculdade de Farmácia, Universidade do Porto, R. Jorge Viterbo Ferreira, nº 228, 4050-313 Porto, Portugal; pandrade@ff.up.pt (P.B.A.); asoliveira@ff.up.pt (A.P.O.); valentao@ff.up.pt (P.V.)

**Keywords:** cytokines, *Homalium tomentosum*, hyaluronan, interleukins, methyl ellagic acid, polyphenols, traditional medicine

## Abstract

Commonly used to treat skin injuries in Asia, several *Homalium* spp. have been found to promote skin regeneration and wound healing. While ethnobotanical surveys report the use of *H. bhamoense* trunk bark as a wound salve, there are no studies covering bioactive properties. As impaired cutaneous healing is characterized by excessive inflammation, a series of inflammatory mediators involved in wound healing were targeted with a methanol extract obtained from *H. bhamoense* trunk bark. Results showed concentration-dependent inhibition of hyaluronidase and 5-lipoxygenase upon exposure to the extract, with IC_50_ values of 396.9 ± 25.7 and 29.0 ± 2.3 µg mL^−1^, respectively. *H. bhamoense* trunk bark extract also exerted anti-inflammatory activity by significantly suppressing the overproduction of interleukin 6 (IL-6) in lipopolysaccharide (LPS)-stimulated RAW 264.7 macrophages at concentrations ranging from 125 to 1000 µg mL^−1^, while leading to a biphasic effect on nitric oxide (NO) and tumor necrosis factor alpha (TNF-α) levels. The phenolic profile was elucidated by HPLC-DAD, being characterized by the occurrence of ellagic acid as the main constituent, in addition to a series of methylated derivatives, which might underlie the observed anti-inflammatory effects. Our findings provide in vitro data on anti-inflammatory ability of *H. bhamoense* trunk bark, disclosing also potential cutaneous toxicity as assessed in HaCaT keratinocytes.

## 1. Introduction

Currently comprising over 70 accepted species listed on The Plant List database (version 1.1 2020; http://www.theplantlist.org), *Homalium* is a genus of deciduous medium-sized trees with a predominant distribution in temperate and subtropical regions [1]. Several members of the genus have been reported due to their use in folk medicine, but data on the pharmacological properties are generally scant. So far, available studies on *Homalium* spp. have demonstrated relevant biological activities frequently related to their ethnomedicinal uses, such as antibacterial [2], antidiabetic [3], antinociceptive [4] and anti-inflammatory [3,4]. 

Ethnobotanical surveys revealed that some *Homalium* species are used as wound healing remedies; *Homalium zeylanicum* Benth. is generally referred to as a wound healing plant [1,5]; leaves from *Homalium foetidum* Benth. are used by tribes from Papua New Guinea to treat topical ulcers and subcutaneous skin infections [2,6]. While there are no available studies on the biological properties of *Homalium bhamoense* Cubbit & W.W.Sm., surveys indicate its utility against common ailments, such as joint pain and fever [1]. Known in Thailand as “kha nang” [7], the roots of the plant are used as an astringent in the neighbouring countries [8], while the leaves and trunk bark are widely used as wound healing remedies also in eastern India [1]. 

Wound healing is an utterly complex process involving an array of finely tuned steps, from initial injury to the final reconstituted tissue, with inflammatory mediators orchestrating a series of events throughout most of the process [9]. For example, the metabolic products of hyaluronidase and 5-lipoxygenase (5-LOX) function as microenvironmental cues, actively participating in the regulation of healing processes and inflammation [10,11]. Nitric oxide (NO) acts as a mediator of skin homeostasis and wound repair, influencing collagen deposition and the strength of incisional wounds [12]. Also cytokines, including tumor necrosis factor alpha (TNF-α) and interleukin 6 (IL-6), act as signalling molecules, being closely involved in the very early events of wound healing through the recruitment of inflammatory cells in the dermal and epidermal layers, synthesis of extracellular matrix proteins and regulation of the immune response [13]. 

A myriad of wound-healing plants have been identified based on ethnobotanical records, a significant number of species used as wound salves in folk medicine exhibiting potent inhibitory effects towards extracellular matrix enzymes. Many species also display ameliorating effects against several additional mediators of inflammation, thus proving to promote the healing rate and reducing scar formation [14]. Worthy of mention is also the increasing number of reports dealing with the development of topical formulations aiming to enhance the anti-inflammatory and skin regeneration properties of herbal extracts [15,16,17], namely nanometric vesicles containing molecules of hyaluronate (hyalurosomes) [16,17].

Due to the scant data on the biological potential of *H. bhamoense*, and also prompted by its ethnomedicinal use, inhibitory effects of a methanol extract obtained from the trunk bark were assessed with regard to a series of inflammatory mediators. Since a persistent inflammatory response derived from skin tissue injury can lead to elevated levels of proinflammatory enzymes and accumulation of free radicals, potential inhibitory properties towards hyaluronidase and 5-LOX were evaluated, as well as the interference with NO levels, both using RAW 264.7 macrophages stimulated with lipopolysaccharide (LPS) and a cell-free assay. The amount of cytokines (TNF-α and IL-6) released by LPS-challenged RAW 264.7 cells was also measured by ELISA. Additionally, the effects upon the viability of the skin representative cell line HaCaT were evaluated in order to deliver data on topical safety. 

Chemical profiling of the extract obtained from the trunk bark was also attained so that one may provide an HPLC fingerprint, also allowing identification of bioactives that may underlie the observed biological effects.

## 2. Results and Discussion

### 2.1. HPLC-DAD Characterization of the Phenolic Profile of H. bhamoense Trunk Bark Extract

Previous studies on the most extensively investigated species, *Homalium ceylanicum* (Gardner) Benth. and *Homalium stenophyllum* Merr. & Chun, have afforded an array of compounds including phenolic acids [18,19,20], isocoumarins [20] and flavonoids [20,21]. However, unlike other *Homalium* species, there are no reports on the chemical profile of *H. bhamoense*. As such, the methanol extract obtained from the trunk bark was investigated in order to deliver an HPLC-based authenticating fingerprint on the plant material, as well as to tentatively identify bioactive polyphenolic constituents. 

**Figure 1:**
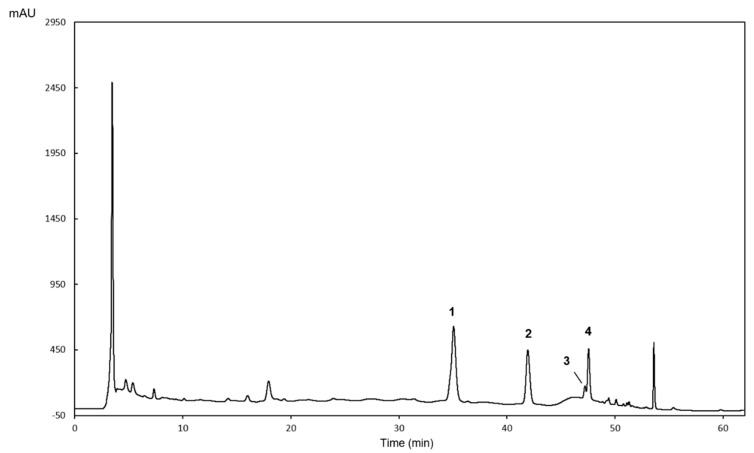
HPLC-UV (252 nm) phenolic profile of the methanolic extract obtained from *H. bhamoense* trunk bark. Identity of compounds as in Table 1.

Comparison with an internal database [22] and authentic standards allowed the identification and quantitation of ellagic acid (**1**), methyl ellagic acid (**4**) and the two glycosylated methyl ellagic acid derivatives **2** and **3** (Figure 1 and Table 1). While the trunk bark extract is predominantly characterized by the presence of methyl ellagic acid derivatives (**2–4**), ellagic acid (**1**) was identified as the main phenolic component, representing ca. 43% of the quantifiable total (Table 1). The phenolic profile of the species is here disclosed for the first time, simultaneously delivering data on the occurrence of ellagic acid derivatives in the genus *Homalium*. 

### 2.2. H. bhamoense Trunk Bark Extract Significantly Inhibits Hyaluronidase and 5-Lipoxygenase

Hyaluronan is a straight-chain glycosaminoglycan carbohydrate polymer detected in all vertebrate tissues, its bulk being produced and deposited in the skin [23]. Its degradation is initiated by hyaluronidases, which leads to the formation of midsized and small fragments having several functional implications, namely acting as distress signals with postulated functions in wound scar formation and accompanying inflammatory responses [12]. 

In most of the mainstay in vitro assays, hyaluronidase obtained from bovine testes is commonly used, since it is commercially available in a reasonably pure form. In contrast with bacterial hyaluronidases, mostly leading to the production of unsaturated disaccharides with limited biological functions, the oligomers resulting from the enzymatic activity of the vertebrates’ homologous display a wide range of size-specific effects [24]. In fact, fold-recognition studies indicate that the catalytic clefts and the active sites are highly conserved between human and bovine hyaluronidases, the latter being structurally representative of all vertebrate hyaluronidases [24]. 

**Figure 2:**
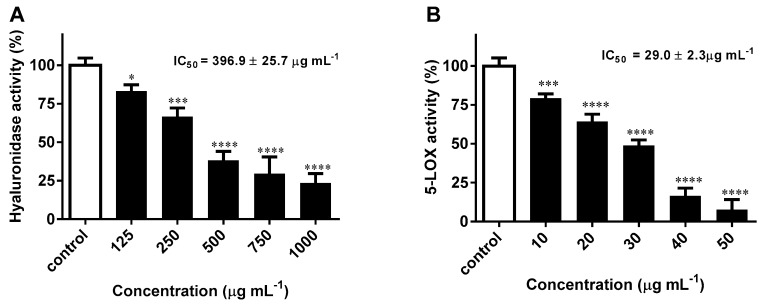
Inhibitory effects towards hyaluronidase (**A**) and 5-lipoxygenase (**B**) activity upon exposure to *H. bhamoense* trunk bark methanol extract. The results correspond to the mean ± SEM of three independent experiments performed in triplicate (statistical significance: * *p* < 0.05, *** *p* < 0.001 and **** *p* < 0.0001).

Our results demonstrate that the methanol extract obtained from *H. bhamoense* trunk bark significantly interferes with the activity of hyaluronidase from bovine testes, at concentrations ranging from 125 to 1000 μg mL^−1^, with an IC_50_ value of 396.9 ± 25.7 μg mL^−1^ being recorded. (Figure 2A). Relevantly, recorded inhibitory effects were stronger than those observed with the well-known inhibitor disodium cromoglycate. As some classes of polyphenols have been described as inhibitors of hyaluronidase, the observed effects might be related to the content in ellagic acid derivatives [25]. In a previous study, Kuppusami and Das [25] noted that non-glycosylated derivatives of ellagic acid were able to cause more than 90% of inhibition. Due to the high content of ellagic acid (**1**) in the methanol extract obtained from *H. bhamoense* trunk bark (Table 1), it seems plausible to consider that the effects may at least partially derive from the inhibitory capacity of this compound. At active concentrations (750–1000 μg mL^−1^) (Figure 2A), the trunk bark extract was found to exhibit a high content in **1**, above the IC_50_ value of 1.5 ± 0.1 μg mL^−1^ recorded by Sgariglia et al. [26]. Partial contribution of **4** cannot be excluded as the methylated derivative is also reported to act as an efficient hyaluronidase inhibitor (IC_50_ = 2.3 ± 0.1 μg mL^−1^) [26].

While hyaluronidases are particularly relevant as enzymatic targets on cutaneous repair, 5-LOX has been increasingly found to play also a critical role. The enzyme catalyzes the initial steps in the conversion of arachidonic acid to leukotrienes [27]. The biosynthesis of lipidic mediators is also related to an increased production of ROS [27] and underlies the inflammatory response in wound healing, with the disruption of 5-LOX being found to improve cutaneous healing [10,28]. Furthermore, classical symptoms of inflammation such as pain, reddening and edema of the surrounding tissue during cutaneous wound healing are caused by the release of eicosanoids [29]. 

While proving to be less effective than the reference inhibitor quercetin (IC_50_ = 8.3 μg mL^−1^), the trunk bark extract led to a significant decrease in 5-LOX activity with concentration dependence (Figure 2B). As seen on Figure 2B, the trunk bark extract proved to be active at concentrations as low as 10 μg mL^−1^, displaying an IC_50_ value of 29.0 ± 2.3 μg mL^−1^. Ellagic acid derivatives, particularly ellagic acid (**1**), have been ascribed as anti-inflammatory agents interfering with several inflammatory mediators [30], and while there are no reports on the specific inhibitory effects towards 5-LOX, Kawakami et al. [31] recorded a concentration-dependent inhibition against the 12-lipoxygenase isoform.

### 2.3. Effects on NO Levels and Inflammatory Cytokines in LPS-Stimulated RAW 264.7 Macrophages

In addition to their phagocytic activity, macrophages play a pivotal role in wound healing, namely in the production and release of mediators involved in the transition from the exudative to the proliferative phase, being the most abundant hematopoietic population in intact skin [13]. NO generally acts as mediator of skin homeostasis and wound repair, influencing collagen deposition and the strength of incisional wounds. However, increased levels lead to an inflammatory response, subsequently activating the synthesis of additional proinflammatory mediators and the proteolytic degradation of extracellular matrix components that dictate the outcome of tissue repair [12,32]. NO release in immune cells, such as macrophages, is activated either through internal stimuli or environmental stressors [33]. For example, NO synthesis is markedly influenced by hyaluronan, its polydisperse fragments mediating inflammatory cell recruitment and promoting the activation of macrophages, namely inducing the expression of inducible nitric oxide synthase (iNOS) and the subsequent production of NO in the early phase of wound healing [32].

**Figure 3:**
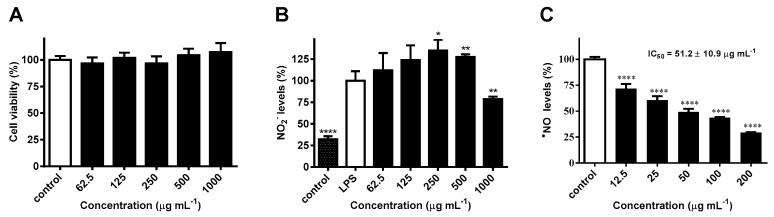
Effects of *H. bhamoense* trunk bark extract on RAW 264.7 cell viability (**A**), NO_2_^−^ levels in lipopolysaccharide (LPS)-challenged RAW 264.7 cells (**B**), and ^•^NO levels in a cell-free assay (**C**). Results correspond to the mean ± SEM of three independent experiments performed in triplicate (statistical significance: * *p* < 0.05, ** *p* < 0.01 and **** *p* < 0.0001).

The nitrite (NO_2_^−^) accumulated in the culture medium of LPS-challenged RAW 264.7 macrophages was used as an index for NO synthesis. Impact on cellular viability was first assessed, results showing no noticeable cytotoxicity after 24 h treatment at concentrations up to 1000 μg mL^−1^ (Figure 3A). Pretreatment with the methanol extract obtained from *H. bhamoense* trunk bark significantly decreased NO levels induced by LPS down to approximately 79% at the highest concentration tested (*p* < 0.01) (Figure 3B). A biphasic effect was observed, as the extract led to a significant increase in NO levels at 250 (*p* < 0.05) and 500 μg mL^−1^ (*p* < 0.01) (Figure 3B). 

The evaluation of antiradical activity in a chemical system showed evidence that the reduction of NO levels might be associated with a pronounced scavenging capacity, as significant effects were observed at concentrations as low as 12.5 μg mL^−1^ (*p* < 0.0001) (Figure 3C). On the basis of the low IC_50_ value of 51.2 ± 10.9 μg mL^−1^ (Figure 3C), the strong scavenging capacity upon ^•^NO might contribute to the observed reduction in NO_2_^−^ levels recorded in the cell-based assay upon exposure to the extract at the highest concentration tested (Figure 3B). 

**Figure 4:**
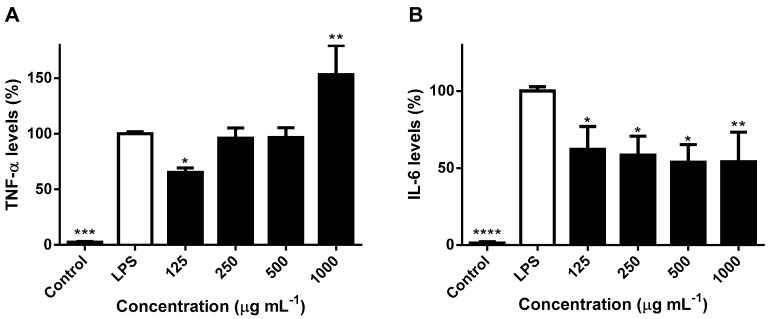
Effects of *H. bhamoense* trunk bark extract on tumor necrosis factor alpha (TNF-α) (**A**) and interleukin-6 (IL-6) levels (**B**) in LPS-challenged RAW 264.7 cells. Results correspond to the mean ± SEM of three independent experiments performed in triplicate (statistical significance: * *p* < 0.05, ** *p* < 0.01, *** *p* < 0.001 and **** *p* < 0.0001).

Activated macrophages also secrete excessive amounts of inflammatory cytokines, such as TNF-α and interleukins, which propagate local inflammation [13]. As such, in addition to NO, production of TNF-α and IL-6 is also frequently employed as criteria to evaluate anti-inflammatory properties, their secretion in the culture of LPS-induced RAW 264.7 macrophages being measured by ELISA assays. The inhibitory effects of *H. bhamoense* trunk bark extract on the proinflammatory cytokines TNF-α and IL-6 are expressed as relative values (measured as pg cytokine/μg of total protein) and are shown in Figure 4. 

A biphasic effect on TNF-α was recorded, characterized by a significant decrease of cytokine levels upon exposure to the extract at 125 μg mL^−1^ (*p* < 0.05) in contrast with a significant increase (*p* < 0.01) at the highest concentration tested (Figure 4A). In alignment with our results (Figure 4A), ellagic acid (**1**) may exert a biphasic effect upon TNF-α in RAW 264.7 cells, as Du et al. [34] reported that it is able to attenuate the cytokine protein levels at concentrations ranging from 1 to 50 µM, but did not prove to impact TNF-α production at concentrations higher than 63 μM [35,36]. According to our results, the significant increase on TNF-α levels at 1000 μg mL^−1^ (*p* < 0.01) might contribute to the wound healing properties of *H. bhamoense* trunk bark, as the cytokine plays a pivotal role in the early phase of wound repair. Increased levels of TNF-α empower the activation of macrophages and acquisition of a proinflammatory M1 phenotype, that enables phagocytosis of microbes and scavenging of dead cells and debris, required for proper cutaneous healing [37]. The effects upon TNF-α synergise the recorded impact of *H. bhamoense* trunk bark towards NO, which has cytostatic, chemotactic and cytotoxic activity against various microorganisms involved in cutaneous infections [38]. TNF-α signalling transiently activates canonical nuclear factor kappa B (NF-κB) target genes, mediating the stimulation of IL-6 production and other cytokines [13,39]. Cutaneous healing is highly regulated by gradients of different cytokines and inflammatory mediators through an intricate and complex network [39]. For example, iNOS is known to be induced by TNF-α, but generated NO may, in turn, modulate TNF-α release. Previous studies demonstrate that NO interferes with cytokine cascades, namely by inducing TNF-α mRNA in RAW 264.7 macrophages [40], neutralization of radical species being also associated with the reduction of TNF-α levels [38].

Compared with the exposure to LPS, the extract obtained from *H. bhamoense* trunk bark proved to significantly reduce IL-6 down to levels ranging from 53.7% to 62.1% at the whole range of concentrations (125–1000 μg mL^−1^) (Figure 4B). While TNF-α induces inflammatory effects by acting directly on multiple target tissues and by inducing other cytokines, such as IL-6 [13], IL-6 also appears to modulate its production. In agreement with our results (Figure 3B and Figure 4), Marcinkiewicz et al. [41] demonstrated that inhibition of IL-6 leads to an enhancement of NO and TNF-α in mouse macrophages. Furthermore, Deakin et al. [42] reported that IL-6 production was attenuated in NO-stimulated mouse macrophages, further suggesting that inhibition of IL-6 by NO may potentiate TNF-α release. The extract obtained from the trunk bark of *H. bhamoense* exerts significant inhibition upon IL-6 levels in LPS-challenged RAW 264.7 macrophages (Figure 4B), suggesting that it may impact the initiation of the cutaneous healing response, as the expression of the effector mediator IL-6 is known to be increased after wounding and persists in older wounds [43]. Ellagic acid derivatives might account to the observed effects, as ellagic acid (**1**) has been reported to significantly decrease IL-6 at the mRNA and protein levels in RAW 264.7 cells stimulated with LPS, at concentrations as low as those found in *H. bhamoense* trunk bark extract at active concentrations (500 and 1000 μg mL^−1^) (Table 1 and Figure 4B) [34,44]. 

### 2.4. Effects in the Viability of Human Keratinocyte HaCaT Cells

Keratinocytes act as the first barrier of protection against external aggressors, being the most common cell type of the human epidermis [45,46]. Besides acting as building blocks of the skin, keratinocytes also play an active role in inflammatory processes, their cellular integrity being essential to attain a proper cutaneous healing [45]. As such, human epidermal keratinocytes are one of the most appropriate cell lines to evaluate skin biocompatibility and cytotoxicity, monolayer cultures of HaCaT cells being one of the most conventional in vitro models to address topical safety [46].

**Figure 5:**
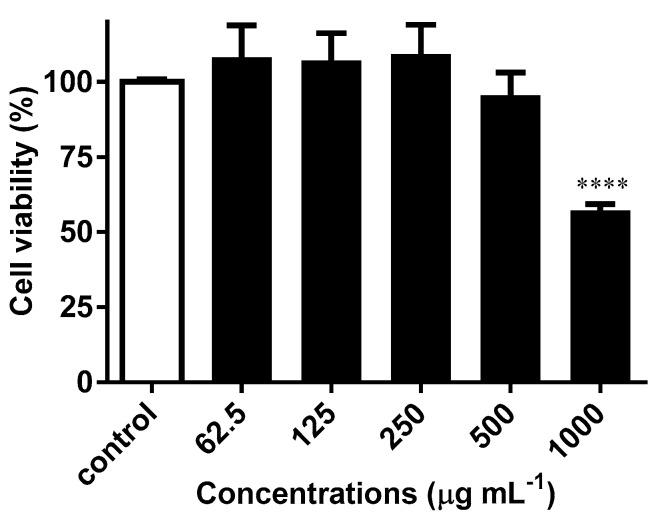
Effects of *H. bhamoense* trunk bark extract on HaCaT cell viability. Results correspond to the mean ± SEM of three independent experiments performed in triplicate (statistical significance: **** *p* < 0.0001).

To determine whether *H. bhamoense* trunk bark extract might exhibit cutaneous toxicity, spontaneously immortalized human keratinocyte HaCaT cells were used as a model. Viability assay testing was attained at concentrations that scored positively in the enzymatic (Figure 2) and cell-based assays (Figure 3 and Figure 4), Figure 5 depicting the extract’s significant cytotoxicity upon 24 h of exposure solely at 1000 µg mL^−1^ (*p* < 0.0001).

## 3. Materials and Methods

### 3.1. General Chemicals and Materials

Methanol p.a. and methanol LiChrosolv^®^ were from Merck KGaA (Darmstadt, Germany). Formic acid was purchased from VWR (Fontenay-sous-Bois, France). 

Purified water was treated in a Milli-Q water purification system (Millipore, Bedford, MA, USA). Spectrophotometric determinations were performed in a Multiskan™ GO microplate spectrophotometer (Thermo Fisher Scientific Oy, Vantaa, Finland).

### 3.2. Collection of Plant Material and Preparation of Crude Extract

Plant material of *H. bhamoense* was collected from Wang Man Sub-district, Wat Sing district, Chainat province, Thailand, in March 2015. The plant was identified and authenticated by Dr. Srunya Vajrodaya, Professor of Botany, Faculty of Science, Kasetsart University, Thailand. A voucher specimen of the plant (PCERU_HB1) was deposited at the Phyto-Chemodiversity and Ecology Research Unit, Faculty of Science, Kasetsart University. Samples were air-dried and ground to a fine powder (mean particle size ≤ 910 μm), 2.45 kg (bark) of powdered material being macerated in 5 L of methanol for seven days, at 28 °C. Resulting extract was filtered through Whatman^®^ grade 1 filtration paper (Sigma Aldrich, St. Louis, MO, USA), concentrated to dryness under reduced pressure in a Büchi Rotavapor^®^ R-210 (Mumbai, India), and stored at −20 °C protected from light, until analysis.

### 3.3. HPLC-DAD Qualitative and Quantitative Analysis

Qualitative and quantitative chromatographic analyses were performed on an analytical HPLC unit (Gilson Medical Electronics, Villiers le Bel, France) coupled with an Agilent 1100 series diode array detector (Agilent Technologies, Waldbronn, Germany). Separation was carried out using a 250 × 4.6 mm, 5 μm, Luna^®^ 100Å C18 column (Phenomenex, Torrance, CA, USA), chromatographic conditions as those described in Ferreres et al. [22]. Briefly, a mobile phase consisting of 1% formic acid in water (A) and methanol (B) was delivered at a flow rate of 0.8 mL min^−1^, starting with 25% B and using a gradient to obtain 35% B at 25 min, 40% B at 40 min, 80% B at 50 min and 80% B at 55 min. The injection volume was 20 μL and the elution was performed at 25 °C. Data processing was performed on Clarity software system 5.04.158 (DataApex Ltd., Prague, Czech Republic). Spectral data were accumulated in the range of 200–700 nm, chromatograms being recorded at 252 nm. Quantitation of the identified compounds was determined from the peak area, using the equation of linear regression obtained from the calibration curve (concentration vs. optical absorbance at 252 nm) (Table 2) built with five concentrations (in triplicate) of ellagic acid (Extrasynthèse S.A., Genay, France).

### 3.4. Enzymatic Assays

#### 3.4.1. Inhibition of Hyaluronidase Activity

Hyaluronidase activity evaluation was carried out spectrophotometrically based on the Morgan–Elson reaction with some modifications [47]. Briefly, 50 µL of plant extract, 50 µL of a stock solution of hyaluronan (HA; 5 mg mL^−1^) and 100 µL of buffer (0.2 M HCOONa, 0.1 M NaCl and 0.2 mg mL^−1^ bovine serum albumin, pH = 3.68) were added to 200 µL of water, reaction being started with the addition of 50 µL of hyaluronidase from bovine testes (600 U mL^−1^) (Type I-S; EC 3.2.1.35; Sigma-Aldrich, St. Louis, MO, USA) prepared in NaCl 0.9%. The reaction was terminated by adding 25 µL of Borax (0.8 M), and subsequent heating for 3 min at 95 °C. The test tubes were cooled and 750 µL of low acidic solution of *p*-dimethylaminobenzaldehyde (DMAB) was added. The tubes were incubated at 37 °C for 20 min and the absorbance was measured at 560 nm. Three independent assays were performed in triplicate, results being compared to the reference hyaluronidase inhibitor sodium cromoglycate.

#### 3.4.2. Inhibition of 5-Lipoxygenase Activity

The potential interference with the enzymatic activity of 5-LOX was determined by the linoleic acid oxidation, according to a previously described procedure [48]. Inhibitory reactions were performed at a final volume of 240 μL, containing 200 μL of phosphate buffer (pH = 9), 20 μL of soybean 5-LOX (EC 1.13.11.12; Sigma-Aldrich, St. Louis, MO, USA) and 20 μL of extract. Preincubation at 25 °C was followed by the addition of linoleic acid (20 μL, 4.18 mM), formation of its oxidation product being followed for 3 min. The absorbance was measured spectrophotometrically at 234 nm, and IC_50_ values were determined against the known 5-LOX inhibitor quercetin. Each measurement was repeated three times, in triplicate.

### 3.5. Nitric Oxide Radical Scavening Activity

Nitric oxide radical scavenging activity was based on the Griess reaction as in Ferreres et al. [49]. A total of 100 µL sodium nitroprusside (10 mmol L^−1^) in phosphate buffer saline (PBS) was mixed with 100 µL of extract, the mixture being further incubated at 25 °C for 60 min under light. After incubation, 100 µL of Griess reagent (1% sulfanilamide, 2% H_3_PO_4_ and 0.1% naphthylethylene diamine hydrochloride) were added, absorbance being measured at 546 nm. Three experiments were performed in triplicate.

### 3.6. Interference with LPS-Induced NO Production by RAW 264.7 Cells

#### 3.6.1. Cell Viability

The effects on cell viability were assessed through the MTT reduction assay [49]. Briefly, RAW 264.7 murine macrophages (ATTC, LGC Standards S.L.U., Barcelona, Spain) were plated in 96-well culture plates at a density of 2.5 × 10^4^ cells well^−1^ and incubated for 24 h in a water-jacket CO_2_ incubator (5% CO_2_, 37 °C; Toreuse model 2428; St. Louis, MO, USA) to reach ca. 80% confluency. The medium was then replaced with 100 μL of plant extract in DMEM and cultured for 24 h. Mitochondrial performance was evaluated through the addition of MTT solution (0.5 mg mL^−1^), the plate being incubated for 90 min at 37 °C. Resulting formazan crystals were dissolved in a mixture of DMSO:isopropanol and absorbance was measured at 560 nm.

#### 3.6.2. Nitrite Assay

Nitrite levels were used as an indicator of the amount of NO, being determined by Griess reagent (1% sulphanilamide and 0.1% *N*-(naphth-1-yl)ethylenediamine dihydrochloride in 2% H_3_PO_4_), as described in Ferreres et al. [49]. Briefly, RAW 264.7 murine macrophages were plated into 96-well plates at a density of 3.5 × 10^5^ cells mL^−1^ and incubated until confluent. Cells were then pretreated with different concentrations of the plant extracts for 2 h and stimulated with LPS (1 μg mL^−1^). Plates were further incubated for 22 h at 37 °C, in a humidified atmosphere of 5% CO_2_, absorbance being read at 540 nm.

### 3.7. Interference with LPS-Induced TNF-α and IL-6 Production by RAW 264.7 Cells

#### 3.7.1. Enzyme-Linked Immunosorbent Assay (ELISA)

The amount of TNF-α and IL-6 released by the LPS-challenged RAW 264.7 murine macrophages was measured by using mouse IL-6 ELISA MAX™ Deluxe and mouse TNF-α ELISA MAX™ Deluxe kits (BioLegend Inc., San Diego, CA, USA) [48]. RAW 264.7 cells were cultured at a concentration of 3.5 × 10^5^ cells mL^−1^ and incubated for 24 h (37 °C, 5% CO_2_). After discarding the medium, *H. bhamoense* trunk bark extract was added to each well at concentrations ranging from 125 to 1000 µg mL^−1^ and incubated for 24 h. Following post-incubation, the cells were treated with LPS (1 µg mL^−1^) for 24 h, culture supernatant being collected and stored at −80 °C until analyzed. 

#### 3.7.2. Total Protein Quantification

Protein concentration was calculated by regression analysis using a standard curve obtained with a solution of bovine serum albumin (BSA), as in Macedo et al. [48]. Colorimetric measurements were attained at 595 nm. 

### 3.8. Interference with HaCaT Cells’ Viability

The effects on the mitochondrial viability of HaCaT cells were assessed through the MTT reduction assay [50]. Briefly, after treatment with 0.25% trypsin solution (GIBCO, Invitrogen, Grand Island, NY, US), human epidermal HaCaT keratinocytes (ATCC^®^) were seeded into 96-well plates at a density of 1.5 × 10^4^ cells well^−1^ in a water-jacket CO_2_ incubator (5% CO_2_, 37 °C; Toreuse model 2428; St. Louis, MO, USA) and grown until reaching ca. 80% confluency after 24h. Then, cells were incubated with 100 μL of plant extract in DMEM GlutaMAX™ and cultured for 24 h. MTT solution (0.5 mg mL^−1^) was added into each well and incubated for 90 min at 37 °C. Subsequently, the MTT solution in each well was removed, with DMSO:isopropanol (3:1) being added. The absorbance was measured at 560 nm. Results correspond to the mean ± SEM of three independent experiments performed in triplicate. 

### 3.9. Statistical Analysis

Statistical analysis was performed by using GraphPad Prism 6.01 software (San Diego, CA, USA). The results of the cell-free experiments were expressed as average IC_50_ values. The results of the experiments were expressed as mean ± SEM, significance between different groups being determined by one-way analysis of variance (ANOVA), followed by Dunnett’s multiple comparison test. *p* values below 0.05 were considered statistically significant.

## 4. Conclusions

In light of the aforementioned information, *H. bhamoense* trunk bark exhibits significant inhibitory properties upon a series of inflammatory mediators involved in wound healing, calling for additional in vitro assays covering cutaneous regeneration as well as the translation to in vivo models of disease. The extract was able to regulate the inhibitory effects of hyaluronidase and 5-LOX in a concentration-dependent manner and may represent one of the cutaneous wound healing mechanisms of *H. bhamoense*. Data from cell-based assays revealed that the methanol extract obtained from the trunk bark of the plant has a biphasic impact on the production of NO and TNF-α in RAW 264.7 macrophages, which seems to be related with its use in folk medicine, namely on the elimination of wound debris and on the inhibition of microorganisms underlying cutaneous infections. However, it is worth emphasizing the effects on the release of the proinflammatory cytokine IL-6, potentially hampering inflammatory processes that delay or impair a normal wound healing.

While additional active components remain to be identified, experimental data suggests that ellagic acid and derivatives might contribute to the recorded anti-inflammatory effects.

Results of this study also have provided scientific basis for the traditional use of *H. bhamoense*, as the trunk bark is expected to accelerate the skin wound healing, as suggested by the enhanced resolution of inflammation in experimental models.

## Figures and Tables

**Table 1:** Content of ellagic acid and derivatives on the methanol extract of *H. bhamoense* trunk bark.

Compound	*R*_t_	mg Kg^−1^ (Dry Extract)
**1** Ellagic acid	34.6	2363.30 ± 53.89
**2** Methyl ellagic acid pentoside	42.4	1590.60 ± 25.51
**3** Methyl ellagic acid deoxyhexoside	47.0	551.52 ± 4.38
**4** Methyl ellagic acid	47.5	1029.36 ± 10.45
**Total**		5534.78 ± 94.23

**Table 2:** Results of linear regression equation analysis, LOD^a^ and LOQ^b^ for ellagic acid.

	Regression Equation					
	Slope (*σ*)	Intercept (*b*)	*R*^2^ (*n* = 3)	Linearity Range (µg mL^−1^)	LOD (µg mL^−1^)	LOQ (µg mL^−1^)
Ellagic acid	164.35	−3443	0.9997	26.88–430	3.55	10.74

^a^ Limit of detection; ^b^ Limit of quantification.

## Abbreviations

5-LOX	5-Lipoxygenase
ATCC^®^	American Type Culture Collection
ELISA	Enzyme-Linked Immunosorbent Assay
iNOS	Inducible nitric oxide synthase
IL-6	Interleukin-6
LPS	Lipopolysaccharide
NF-κB	Nuclear factor kappa B
NO	Nitric oxide
TNF-α	Tumor necrosis factor α
